# Highly parallel simulation and optimization of photonic circuits in time and frequency domain based on the deep-learning framework PyTorch

**DOI:** 10.1038/s41598-019-42408-2

**Published:** 2019-04-11

**Authors:** Floris Laporte, Joni Dambre, Peter Bienstman

**Affiliations:** 1 Photonics Research Group, UGent - imec, Technologiepark-Zwijnaarde 126, 9052 Ghent, Belgium; 2 IDLab, UGent - imec, Technologiepark-Zwijnaarde 126, 9052 Ghent, Belgium

## Abstract

We propose a new method for performing photonic circuit simulations based on the scatter matrix formalism. We leverage the popular deep-learning framework PyTorch to reimagine photonic circuits as sparsely connected complex-valued neural networks. This allows for highly parallel simulation of large photonic circuits on graphical processing units in time and frequency domain while all parameters of each individual component can easily be optimized with well-established machine learning algorithms such as backpropagation.

## Introduction

Although photonic circuit simulation software already exists, it definitely has not yet reached the same maturity as electronic circuit simulation software. This is mostly due to the complication of having both amplitude and phase modulation in each component, which makes it very difficult to predict the behaviour of large circuits with many components due due to interference effects. Optimizing photonic circuits has thus long been a process of trial and error, where several parameters are swept independently of each other.

As of 2019, there are a handful of simulators for designing photonic integrated circuits, such as Aspic^1^, Luceda Caphe^2^, Lumerical Interconnect^3^ and VPI Photonics^4^. All are excellent circuit simulation tools for their particular purpose. However, some of these photonic circuit simulation tools are not well suited for parallel simulations and for many of them, optimizing a circuit means nothing more than just sweeping the parameters, which quickly becomes unwieldy when the number of parameters or components in the circuit starts to grow.

To address this, we present Photontorch, a tool loosely based on the node-based approach of Caphe^2^, which in itself is based on coupled-mode theory^5,6^, but reduces the number of parameters by eliminating memory-less nodes that are independent on time before doing a simulation. Our tool is written in Python and uses PyTorch tensors^7^ to describe the parameters and S-matrices of the components. PyTorch tensors are highly optimized arrays, which, as opposed to the more commonly used Numpy ndarray^8^, can be placed on the Graphical Processing Unit (GPU) of a computer, automatically enabling highly parallelizable simulation of photonic circuits simulations. Moreover, each operation done on these tensors also tracks the gradient of that operation on the result, enabling backpropagation^9^, currently the default optimization method for deep neural networks with thousands of parameters.

The similarities with photonic circuits and neural networks are not a coincidence. Just like in neural networks, most of the actions of a photonic circuit can be described by linear matrix algebra. Sometimes however, an active component changes the behaviour of the circuit in a non-linear way, which can be compared to applying a custom activation function in the field of neural networks. It is this approach of treating a photonic circuit as essentially a sparsely connected recurrent neural network that may be a key ingredient in future photonic circuit design.

The remainder of this paper is organized as follows. First, a Description of the framework is given: its core components and the equations that govern them are described. Next, a short comparison with other photonic simulators is given in terms of performance on the core application domain of Photontorch: parallel multi-modal simulation of multiple input signals through large photonic circuits. Finally, the advantages of optimizing photonic circuits through backpropagation are showcased by providing three examples: one in the frequency domain and two in the time domain.

## Description of the framework

### Components

To design a circuit in Photontorch, one needs *components* as building blocks. Each component consists of *N* ports and each of these ports can be related to any other port of the same component by a *scatter*-matrix or S-matrix, for which each element *S*_*ij*_ describes the instantaneous connection between port *i* and port *j*.

Furthermore, each component keeps track of which of its ports act actively. Active - or more generally memory-containing (MC) Caphe^2^ - ports are for example source ports, detector ports, ports introducing optical delay or ports that are defined by a custom action *f*_act_, such as a Semiconductor Optical Amplifier (SOA), where the current action depends on an internal state. In general, any input state *x*_in_ at time *t* gets transformed to an output state *x*_out_ at time *t* as follows:

1 $${x}_{{\rm{out}}}(t)=S\cdot {x}_{{\rm{in}}}(t)+{f}_{{\rm{act}}}(t,x(t),x(t-dt),\ldots )$$

### Networks

Multiple components can be combined in a circuit or *network* by combining the individual component S-matrices into a joined *block-diagonal* S-matrix. The connections between the components can be represented by a *connection* matrix or C-matrix with elements *C*_*ij*_ ∈ {0, 1}, effectively mapping the output state *x*_out_ back onto an input state *x*_in_:

2 $${x}_{{\rm{in}}}(t+dt)=C\cdot {x}_{{\rm{out}}}(t)$$

A Photontorch network is in itself also a Photontorch component, allowing for a very hierarchical structure while defining a top-level network. A network is a *top-level* network when it is *fully connected*, i.e. when

3 $$\sum _{i}{C}_{ij}=\sum _{i}{C}_{ji}=\mathrm{1\ \ \ }\forall j\mathrm{.}$$

This network cannot be connected to other components, as it has no free ports left.

### Reduced connection matrix

For these kind of top-level networks, the number of ports can be reduced. This is done by combining (1) and (2) into

4 $$(\begin{array}{c}{x}^{ml}\\ {x}^{mc}\end{array})=(\begin{array}{cc}{C}^{ml,ml} & {C}^{ml,mc}\\ {C}^{mc,ml} & {C}^{mc,mc}\end{array})\cdot (\begin{array}{cc}{S}^{ml,ml} & 0\\ 0 & {S}^{mc,mc}\end{array})(\begin{array}{c}{x}^{ml}\\ {x}^{mc}\end{array})+(\begin{array}{c}0\\ {f}_{{\rm{a}}{\rm{c}}{\rm{t}}}({x}^{mc})\end{array}),$$

where the division was made between instantaneous memory-less (ML) ports and memory-containing (MC) ports. The ML part of the equation is independent of time and can be inverted^2^ to define a reduced equation, describing only the action on the MC ports:

5 $${x}^{mc}(t+dt)=({C}^{mc,mc}+{C}^{mc,ml}\cdot {S}^{ml,ml}\cdot {(1-{C}^{ml,ml}{S}^{ml,ml})}^{-1}{C}^{ml,mc}){S}^{mc,mc}{x}^{mc}(t)+{f}_{{\rm{a}}{\rm{c}}{\rm{t}}}({x}^{mc})$$

6 $$=\tilde{C}{S}^{mc,mc}{x}^{mc}(t)+{f}_{{\rm{a}}{\rm{c}}{\rm{t}}}({x}^{mc}),$$

where $\tilde{C}$ is the reduced C-matrix, which describes all the instantaneous connections. In the following, the superscripts will be dropped, assuming that the reduction is already performed and only the MC nodes are left.

Ports for which a custom action is defined will have access to the state *x*, as well as previous states through a buffer. This allows for example the definition of a component action in the form of an ordinary differential equation (ODE).

### Parallelized design

An important novelty of the Photontorch framework compared to commercially available software^1–4^ is the fact that the state vector *x* is defined as a big monolithic multidimensional tensor. Its dimensions describe, apart from the number of MC nodes, the different wavelengths (or modes) of the simulation and the different parallel simulations (batch size). The S-matrix and C-matrix have an extra dimension for each wavelength as well, which allows for very fast simultaneous simulation for multiple wavelengths at once on a GPU. In the time domain, this parallelizable nature is even more clear, as one can parallelize both the number of input wavelengths and the number of input wave forms. The parallelized version of the update equations (6) for a network with *N* memory containing nodes can be written as:

7 $${x}_{(q+\mathrm{1)}mnb}=\sum _{i}^{N}\sum _{j}^{N}{C}_{mni}{S}_{mij}{x}_{qmjb}+{f}_{{\rm{a}}{\rm{c}}{\rm{t}}}(t,{x}_{qmnb},{x}_{(q-\mathrm{1)}mnb},\ldots ),$$

where *q* represents the current time step such that *x*(*q* ⋅ *dt*) = *x*_*q*_, *m* represents the number of wavelengths (or modes), *n* represents the number of MC nodes and *b* represents the number of parallel simulations performed at once, i.e. the *batch size*.

### Circuit optimization

Photontorch is entirely written with a PyTorch^7^ backend. PyTorch is a popular *deep-learning* framework designed to optimize large tensor networks with backpropagation. By writing the Photontorch components in terms of optimizable PyTorch parameters, PyTorch will automatically keep track of the necessary gradients to perform backpropagation through the circuit. This enables a whole new way of optimizing the parameters of the photonic circuit.

## Performance metrics

Although performance was never the main objective for Photontorch, the parallel nature in which it was built up allows for very efficient execution in certain cases, especially for *passive circuits* in the time domain.

**Figure 1:**
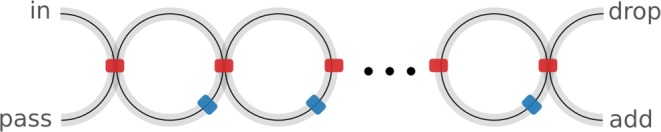
A CROW is an add-drop filter with extra rings. Each CROW with *n* rings has *n* + 1 couplings (blue) and *n* phase shifts (orange).

As an example, the performance of Photontorch was bench-marked by simulating a Large Coupled Resonator Optical Waveguide (CROW) both in the frequency domain and in the time domain. A CROW consists of a number of ring resonators which are connected to each other in series, as can be seen in Fig. 1. Such A CROW is a good circuit for bench-marking, as it allows to easily add extra rings to increase the difficulty of the simulation. Other parameters that can be tweaked during a CROW circuit simulation are the number of wavelengths simulated simultaneously and the number of parallel simulations performed at the same time, in a batched execution mode. All simulations were performed on a normal desktop computer with an Intel i7-4790K CPU with 8 GB RAM, while for the GPU simulations, an Nvidia GTX-1060 (6 GB) GPU was used. The performance of Photontorch (both CPU and GPU) on simulating this passive circuit is compared with other simulators, such as *Lumerical Interconnect* and *Luceda Caphe*.

**Figure 2:**
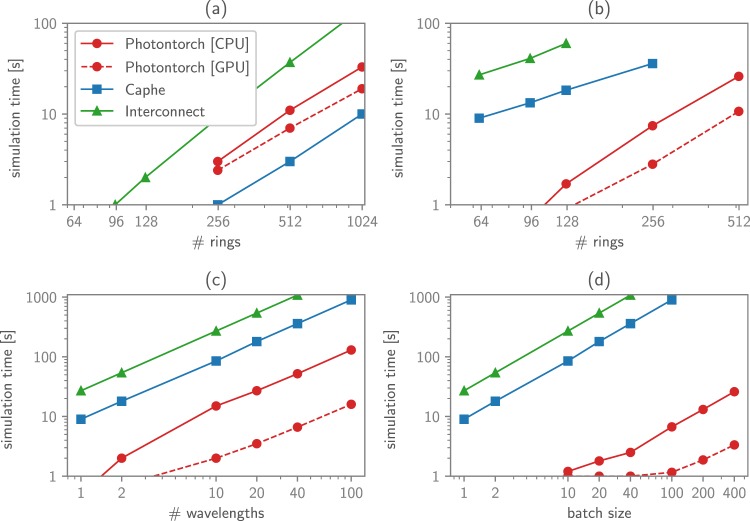
The performance for Photontorch simulating a CROW, both in the frequency domain and the time domain, was compared to *Interconnect* and *Caphe*. (**a**) The time needed to find the frequency response for a CROW of increasing number of rings: the performance of Photontorch lies somewhere in between the Caphe and Interconnect. (**b**) The time needed to do a time domain domain simulation of 3000 time steps for an increasing number of rings: the simulation time of Photontorch is practically zero up to about 100 rings. (**c**) Performance for a multimode time-domain simulation for a CROW of 64 rings and an increasing number of wavelengths. (**d**) Performance for a time-domain simulation of a CROW with 64 rings for a single wavelength but for an increasing number of input waveforms (batch size).

First, the response of a CROW in frequency domain was calculated. For this task, Photontorch is outperformed by Caphe, but performs significantly better than Interconnect, as can be seen in Fig. 2(a). Caphe performs better in this regard due to its more efficient solver for a large system of equations necessary to find the reduced connection matrix of the CROW. This solver utilizes a factorization method for sparse systems^2^, which is currently not available in Photontorch’s PyTorch backend, but could conceivably be added.

However, once the S-matrix is found, Photontorch vastly outperforms both Caphe and Interconnect in time-domain simulations of the CROW, as can be seen in Fig. 2(b–d), where a CROW was simulated for 3000 time steps. Indeed, in Fig. 2(b), one sees that Photontorch outperforms both Caphe and Interconnect for a time-domain simulation of a CROW with an increasing number of rings. Moreover, simulating additional wavelengths at once for a CROW with 64 rings is always faster than the sequential simulation required by Caphe and Interconnect, as can be seen in Fig. 2(c). Similarly, simulating multiple input wave forms at once (batched execution) for a CROW with 64 rings at a single wavelength generates almost no overhead in Photontorch, especially on a GPU Fig. 2(c).

## Optimization results

Apart from its parallel nature, Photontorch can also be used to efficiently optimize large photonic circuits through backpropagation. Backpropagation is a well-established optimization method which is traditionally used to optimize the many parameters of deep neural networks^10^. Since the Photontorch framework exclusively uses PyTorch’s autograd tensors and operators^7^, each operation for which a forward pass is defined will be differentiable. This allows us to optimize complex photonic circuits as if they were recurrent neural networks. This way of optimizing photonic circuits is a lot more efficient than sweeping the parameters of the circuit or optimizing through genetic algorithms, as a much smaller portion of the parameter space has to be explored.

Photonic circuits are typically recurrent in nature, which will have an effect on how effective backpropagation is, as exploding gradients and vanishing gradients are common problems for large recurrent neural networks^11^. In deep learning these problems are often solved by using specialized recurrent modules such as the well-known Long Short-Term Memory (LSTM) cell^12^ or the Gated Recurrent Unit (GRU)^13^. However, recent advances have shown that recurrent deep learning with unitary matrices^14,15^ does not suffer from this problem. Loss-less photonic components are per definition unitary, which will allow us to still find a suitable optimum for many circuit optimization problems through backpropagation. In the case of lossy structures, the losses are typically low enough to consider the photonic circuit quasi unitary.

### Coupled Resonator Optical Waveguides

**Figure 3:**
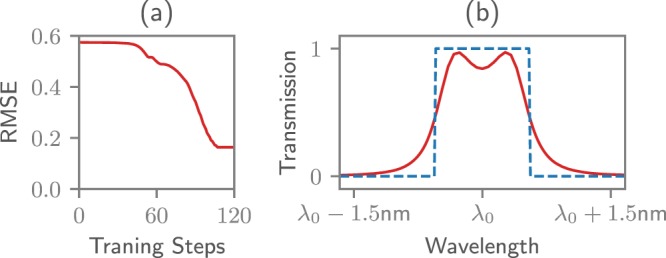
The parameters for a CROW-based band-pass filter can easily be obtained through backpropagation. (**a**) The evolution of the Root Mean Squared Error (RMSE) between target function and transmission at the drop port of the CROW over time. Each training step takes about 100 ms. (**b**) The transmission at the drop port was trained to create a window of transmission of 1 nm around a center wavelength *λ*_0_ = 1545 nm.

First of all, the same CROW as used for the performance measurements was optimized in the frequency domain to act like a band-pass filter around *λ*_0_ = 1545 nm. A CROW of 4 rings was assumed, each with a radius of 5 *μ*m. The only parameters being optimized are the couplings between the rings. After a few training steps, an optimum is found through gradient descent, as can be seen in Fig. 3(a). The resulting transmission at the drop port of the CROW approximates the target transmission window of 1 nm width as can be seen in Fig. 3(b). The whole training procedure was finished in a few seconds.

### Passive Photonic Reservoir Computer

**Figure 4:**
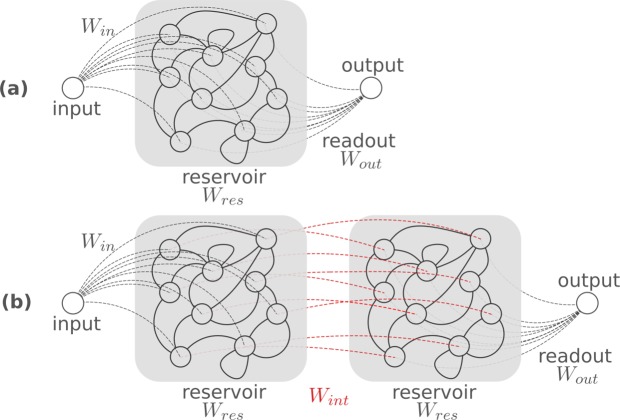
(**a**) A single-input reservoir computer. A single input is distributed over the nodes of a reservoir by a fixed set of input weights *W*_*in*_. The reservoir has a complex recurrent interconnection topology characterized by its intermediate weights *W*_*int*_. The reservoir states are read out by a trainable set of readout weights *W*_*out*_. (**b**) An ensemble of reservoirs. Two reservoirs are cascaded by a trainable set of intermediate weights *W*_*int*_.

Furthermore, it is shown that such optimization through backpropagation can also be applied in the time domain. To illustrate this, a large photonic reservoir^16^ was optimized. Reservoir computing is an almost two-decade-old machine-learning concept^17,18^. It is defined by distributing an input signal over a series of nodes which are *recurrently* connected, as shown in Fig. 4(a). The connections between the recurrent nodes are not optimized and form the so-called *reservoir*. In fact, only the output connections that combine the states in the recurrent nodes into a useful output signal are optimized for the task at hand. The reservoir is called *passive* if no non-linearities are present inside it. Such passive reservoirs rely solely on the non-linear operation at the photodetector and can easily be implemented in photonic circuits with splitters and combiners^16^.

In most on-chip reservoirs, the reservoir states are first detected before they are linearly combined into an output signal (the so-called electrical readout). Although this first-detect-then-weight approach produces good results, it is not very feasible for large reservoirs, as one would need as many detectors as there are reservoir nodes. On top of that, using multiple detectors and analog-to-digital converters goes against the idea of having an energy-efficient system. For an all-optical implementation, it is beneficial to move the detectors at the nodes of the reservoir to one single detector at the output, after an optically implemented weighting procedure, implemented e.g. by amplitude and phase modulators (a so-called optical readout). These complex-valued readout weights should be trained to minimize the Mean Squared Error (MSE) between the detector output and the target signal.

The traditional approach to train reservoirs uses ridge regression^19^ to optimize a real-valued sum with real-valued weights^20^. While it is possible to use a complex extension of this to optimize a complex-valued sum with complex-valued weights, this is not entirely what is needed in order to train the optical readout. Indeed, one only cares about the amplitude of signal after the detector, whereas complex-valued ridge regression would only be able to aim for a given complex summed signal before the detector. However, there are many different complex-valued signals (each with a different phase) before the detector that give rise to the same intensity after the detector. In order to be able to use ridge regression, the phase of the signal in front of the detector would need to be arbitrarily fixed, effectively limiting the space of complex optical weights.

This is where Photontorch can be of invaluable help, as it enables to perform backpropagation through the detector without having to make any assumption on the phase before the detector. This technique can also be used in the case of the cascaded reservoir in Fig. 4(b), where using complex ridge regression would be completely out of the question.

To see how Photontorch can be used in this case, a reservoir was trained to perform the XOR task: a Pseudo Random Bit Sequence (PRBS) of 10^5^ bits was sent through the reservoir, while the readout was trained to calculate the XOR on two bits in the stream. Both a single reservoir of 36 nodes and a cascaded reservoir of two times 18 nodes were trained.

Throughout the simulations, a detector with load resistance *R*_*L*_ = 1 kΩ, responsivity *η* = 0.5 *A*/*W* and frequency cut-off *f*_*c*_ = 50 G*bps*, implemented by an order-4 Butterworth filter was assumed. The detector noise consists of thermal noise and shot noise, respectively modelled by a Nyquist process and a Poisson process.

**Figure 5:**
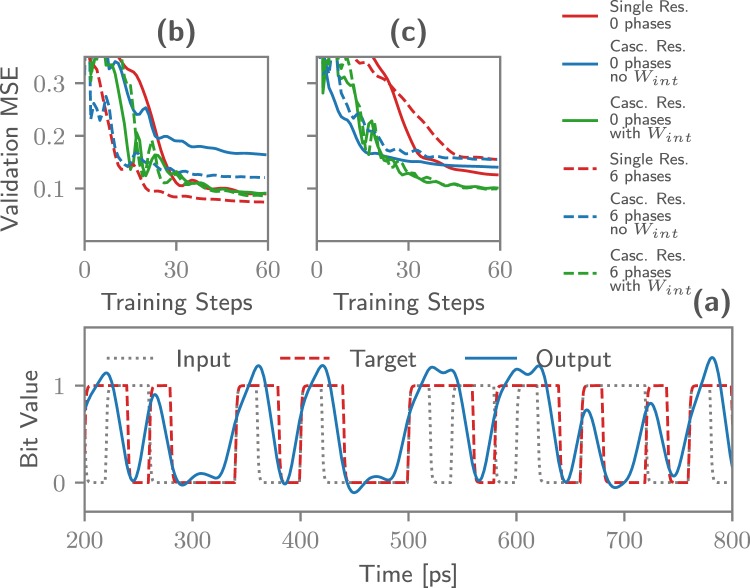
(**a**) Learning curve depicting the validation error between on the XOR task on two subsequent bits. By allowing a few phases inside the reservoir to vary, the MSE can be improved. These optimized phases are traditionally very difficult to find using conventional simulation techniques. (**b**) Learning curve depicting the validation error on the XOR task on two bits with one bit in between. This is more easily performed by the cascaded reservoir. However, better performance is only found with the cascaded reservoir by training the intermediate weights *W*_*int*_. (**c**) Sample output stream for the reservoir with 2 phases optimized for the XOR on two subsequent bits. Good correspondence between the target stream and the reservoir output is observed.

As a first task, the XOR on two subsequent bits in the bit stream was performed, shown in Fig. 5(a). The output weights of both reservoirs and the intermediate weights *W*_*int*_ of the cascaded reservoir were trained to return a signal that resembles the target signal as close as possible. For this, backpropagation through the detector (and through the second reservoir in the case of the cascaded reservoir) was used to minimize the MSE between the target signal and the output signal. In Fig. 5(b) one sees that the normal reservoir and the cascaded reservoir for which the intermediate weights *W*_*int*_ are optimized perform equally well on this task, while the cascaded reservoir without intermediate weights performs noticeably worse. However, if a few connection phases in the normal reservoir were allowed to be tuned as well, the MSE can be decreased even further, even without needing to cascade two reservoirs.

As a second task, the XOR on two bits with one bit in between is taken as a target. As can be seen in Fig. 5(c), initially the cascaded reservoir for which the intermediate weights *W*_*int*_ are being trained performs worse than the normal reservoir when only the readout weights are optimized. However, its performance can be improved if one allows optimization of the intermediate weights *W*_*int*_.

### Unitary matrices

Finally we show that Photontorch can also be used to optimize optically implemented unitary matrices^21–23^. By training a network of cascaded optical mixing units to perform the permuted pixel-by-pixel MNIST (Modified National Institute for Standards and Technology) digit recognition task^15,24^.

Implementing and tuning such large networks on chip is currently still in a very early stage^25^, however, insights from these endeavours have already lead to new deep-learning architectures^15^, which outperform more traditional Long Short-Term Memory (LSTM) cells^12^ on several benchmark tasks, as they do not suffer from common problems in recurrent neural networks, such as exploding or vanishing gradients^11^. More recently, more efforts have been made to streamline the initialization and optimization process of these large networks using backpropagation^26^. Having a tool like Photontorch might help significantly for prototyping those photonics-inspired neural networks.

**Figure 6:**
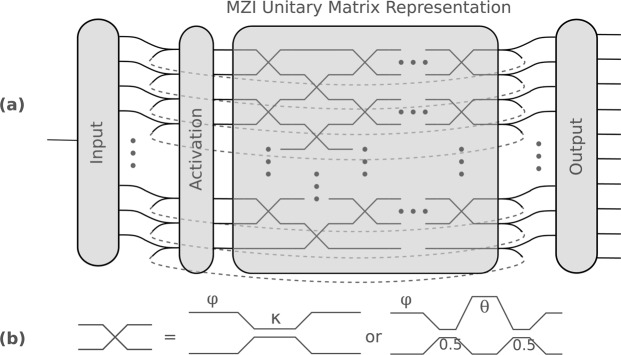
(**a**) Any unitary matrix can be represented by a cascade of mixing units. To span the full unitary matrix space, the number of mixing unit layers needs to be equal to the rank of the matrix to represent. By looping the unitary matrix onto itself, one gets a unitary recurrent neural network. The network represented here contains an input layer, which transforms the 1D time dependent input data to a 256D. This state then gets sent through the unitary matrix, which is connected onto itself. The output weights transforms the recurrent layer back into a 10D state, one output for each digit to recognize. To boost the power of the recurrent neural network, an activation or non-linear element has to be added into the recurrent loop. (**b**) The photonic mixing unit used to build the unitary matrix needs to possess two independent variables. It can either be represented by a phase shift followed by a directional coupler with variable coupling or by an MZI containing two phase shifts.

Implementing those unitary matrices in photonics is in theory quite trivial. Since lossless optical components are per definition unitary, those unitary matrices can be constructed by cascading layers of mixing units together as illustrated in Fig. 6(a). Such a mixing unit can be any 2 × 2 port for which the phase and the coupling can be controlled, such as a directional coupler preceded by a phase shifter or a Mach-Zehnder interferometer (MZI) with two variable phases, as illustrated in Fig. 6(b). In the following, layers consisting of MZIs were assumed.

The number of such MZI layers, often called the *capacity* of the network, is a free parameter of the system. It turns out that one needs a *full-capacity* network to span the full unitary matrix space, i.e. the number of MZI layers needs to be equal to the rank of the unitary matrix. However, networks with less capacity can also be used with great results in the permuted pixel-by-pixel MNIST of recurrent neural networks^15^.

Such a unitary recurrent neural network (URNN) is easily formed by connecting the output of the unitary matrix back onto itself, as illustrated in Fig. 6. It is such a URNN that was chosen to perform the permuted pixel-by-pixel MNIST task, a common benchmark task for recurrent neural networks, where one tries to perform digit recognition on an image that is sent pixel-by-pixel through the neural network in a fixed randomized order^14,15^.

The architecture of the URNN, illustrated in Fig. 6, is defined as follows. A single input (which will take the image pixels one by one), gets transformed into a 256D state by an array of optimizable weights. This 256D state gets then fed into the unitary matrix network of capacity 3, i.e. in three layers of each 128 MZIs (each MZI has two inputs). The outputs of this EURNN get split: one part gets fed into the output layer and one part gets sent back to the input of the unitary matrix. The output layer in itself is again an array of 10 × 256 weights, which makes a linear combination for each of the possible digit responses. The output number with the largest resulting amplitude is the answer of our network.

The input and the output layer can in principle be represented by a photonically implemented unitary matrix as well, but we chose not to do this as to not make the model overly complex. The total number of parameters represented by the cascade of MZIs, is 2 × 128 × 3 = 768, as each MZI contains two optimizable parameters: the input phase difference *ϕ* and the phase difference between its arms *θ*. Optimizing this many parameters with a conventional circuit simulator would be a nightmare.

To boost the performance of the network defined above, a non-linear layer has to be added to the recurrent loop. This non-linear element was implemented in simulation by the modrelu^15^ function. However, Photontorch allows in principle to easily swap out this non-linearity for a more physically achievable non-linearity, for example implemented by a Semiconductor Optical Amplifier (SOA).

**Figure 7:**
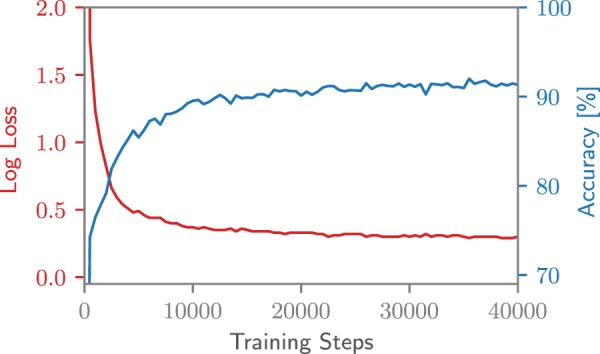
Learning curve for the pixel-by-pixel MNIST task with a capacity-3 unitary neural network.

The final accuracy on the MNIST digits for the permuted pixel-by-pixel MNIST task is 92%, as can be seen in Fig. 7. This is on par with previously documented results for unitary matrices^14,15^. However, in this case, the core of the network was defined solely using Photontorch components, which makes it a very modular approach. This allows for example to change the network at certain locations by changing some of the MZIs by more complex components. Moreover, the Photontorch framework allows to easily experiment with completely different photonics-inspired neural network designs that are less easily implemented with conventional modelling tools.

## Discussion

The presented framework adds a new approach to the not yet completely mature photonic circuit simulation landscape in two major ways: it facilitates the simulation of large photonic circuits in a parallel way and more importantly, it introduces a completely new way of optimizing photonic circuits.

We demonstrated by providing concrete examples that this deep-learning based photonic circuit simulator can be of great value to optimize photonic circuits. The inherent parallelism and interference effects of photonics makes optimizing circuits a lot harder than for example in electronics; it is thus completely normal to suspect that other approaches are needed for optimizing large photonic circuits with a large number of parameters.

The proposed simulator Photontorch shows a lot of promise for such photonic circuit simulation and optimization. It is an ideal choice when simulating a passive circuit for multiple wavelengths in the time domain. Additionally, the inherent parallel nature also allows to simulate the batched response to different independent input waveforms simultaneously at almost no overhead.

The main feature of Photontorch is its inherent relation to PyTorch autograd tensors, allowing it to leverage backpropagation through each photonic component to optimize the parameters of large photonic circuits. We expect this to be incredibly useful for prototyping photonic circuits, as well as for optimizing the parameters in arbitrary photonic circuits containing both passive and active elements, such as - but definitely not excluded to - the large MZI network discussed in this paper.

This feature might act as a double-edged sword, however, as having to describe each operation in terms of differentiable PyTorch tensors inherently limits what kind of computations can be done efficiently, while in addition, GPUs generally are more efficient for linear operations. This means that - although Photontorch is certainly capable of doing so - circuits with many active components will not be simulated as efficiently as the highly optimized CPU-code found in some other simulators.

Finally, we also expect this framework to be useful for prototyping optically inspired neural networks for machine learning. The way of defining a network as essentially linked components or modules through a connection matrix can possibly unlock architectures that are presently hard to describe with more conventional deep-learning methods.

## Data Availability

The MNIST Dataset can freely be downloaded from: http://yann.lecun.com/exdb/mnist. The scripts used to obtain the performance metrics and optimization results can be found on GitHub: http://github.com/flaport/photontorch_paper.
